# First-in-human application of decellularized human amniotic membrane hydrogel as an injectable intraocular scaffold for large idiopathic full-thickness macular hole closure: four-month clinical outcomes

**DOI:** 10.1186/s40942-026-00893-4

**Published:** 2026-06-24

**Authors:** Jakkrit Juhong, Hathaichanok Impheng, Mukdawan Sukhang, Auemphon Mordmuang

**Affiliations:** 1 Department of Ophthalmology, Walailak University Hospital, Walailak University, Nakhon Si Thammarat, 80160 Thailand; 2 Department of Medical Sciences, School of Medicine, Walailak University, Nakhon Si Thammarat, 80160 Thailand; 3 Department of Physiology, Faculty of Medical Science, Naresuan University, Phitsanulok, 65000 Thailand; 4 Institute of Pathology, Ministry of Public Health, Bangkok, 10400 Thailand

**Keywords:** Macular hole, Amniotic membrane hydrogel, Decellularized ECM, Vitrectomy, ILM peeling, mfERG, Intraocular scaffold, First-in-human

## Abstract

**Background:**

Large idiopathic full-thickness macular holes (FTMH) with minimum diameter > 400 μm carry surgical success rates of approximately 60% with standard vitrectomy and gas tamponade. Novel biologic scaffolds have improved closure rates but are limited by intraoperative handling challenges. We describe a surgical technique using thermosensitive injectable decellularized human amniotic membrane (dAM) hydrogel as an intraocular scaffold for large FTMH, and illustrate its feasibility and safety in a first-in-human application with four-month anatomical, functional, and electrophysiological outcomes.

**Surgical technique:**

A 62-year-old man with a Gass stage 4 idiopathic FTMH (minimum diameter 572 μm) and preoperative BCVA of 20/200 underwent combined phacoemulsification and 25-gauge pars plana vitrectomy (PPV) with ILM peeling. Following fluid-air exchange, dAM hydrogel (10 mg/mL, thermosensitive, prepared by detergent decellularization and pepsin solubilization) was injected via a 25-gauge soft-tip cannula, followed by SF6 gas tamponade. Pre-clinical biocompatibility was confirmed by CCK-8 assay and transwell invasion assay prior to clinical use.

**Outcomes:**

At one month, SD-OCT confirmed complete Type 1 anatomical closure and BCVA improved to 20/80. At four months (day 121), BCVA further improved to 20/40 + 1 (logMAR 0.30), with no metamorphopsia, IOP 12.7 mmHg, and sustained Type 1 closure with progressive ellipsoid zone (EZ) reconstitution on OCT. Multifocal ERG (103 segments) confirmed measurable foveal P1 responses (Ring 1: 34.19 nV/deg²) with normal central N1 implicit time (40.2 ms; normal range 36.8–46.4 ms). No intraocular adverse events occurred at any time point.

**Conclusions:**

This injectable dAM hydrogel scaffold technique is a feasible, instrument-sparing method for large FTMH repair, achieving stable Type 1 closure with progressive visual and electrophysiological recovery over four months. mfERG evidence of preserved foveal photoreceptor function supports its safety. Prospective comparative evaluation is ongoing (ClinicalTrials.gov: NCT06433284).

**Clinical trial registration:**

ClinicalTrials.gov NCT06433284 (MACROHOLE trial). Registered April 2024.

## Background

Idiopathic full-thickness macular hole (FTMH) affects approximately 33 per 10,000 individuals older than 55 years [1]. While standard 25-gauge pars plana vitrectomy (PPV) with internal limiting membrane (ILM) peeling and gas tamponade achieves closure rates exceeding 90% for holes with minimum diameter (MD) < 400 μm, success drops to approximately 60% for large holes with MD > 400 μm [2, 3].

Adjunctive techniques for large FTMH include the inverted ILM flap (IFT) [4] and the human amniotic membrane (hAM) plug [5]. The hAM plug leverages the anti-inflammatory, [6] anti-fibrotic [7], and low-immunogenicity properties of amniotic tissue, achieving 100% anatomical success at six months [5]. However, intraoperative handling of solid hAM sheets is challenging: sub-millimetre trimming is imprecise, tissue loss through vitrectomy ports is possible, and precise intravitreal placement is technically demanding [8].

Converting decellularized amniotic membrane (dAM) into a hydrogel form addresses these limitations. Decellularization preserves the extracellular matrix (ECM) scaffold—collagen, laminin, fibronectin, and bioactive growth factors—while removing immunogenic cellular components [9]. The resulting thermosensitive hydrogel is injectable through standard 25-gauge cannulas, conforms to the geometry of large holes, and gels in situ at 37 °C. Although dAM hydrogels have been explored in wound healing, cardiac, and cartilage repair, intraocular application has not been previously reported [9].

We describe an injectable dAM hydrogel scaffold technique for large FTMH—including hydrogel preparation, pre-clinical characterization, and the step-by-step intraocular delivery method—and illustrate its feasibility, safety, and four-month anatomical, functional, and electrophysiological outcomes in a first-in-human application.

## Surgical technique and illustrative case

### dAM hydrogel preparation and pre-clinical characterization

Human amniotic membranes were obtained from donated placentas following elective cesarean sections at Walailak University Hospital, with full informed consent and serological screening for HIV, hepatitis B, hepatitis C, and syphilis. Membranes were aseptically separated from the chorion, washed with PBS containing 5% penicillin/1% streptomycin, and enzymatically decellularized using 10% trypsin followed by extensive PBS washing. Sterilization was performed with peracetic acid. Membranes were stored at − 80 °C until use.

For hydrogel preparation, dAM was mechanically homogenized in PBS and solubilized with pepsin (1 mg/mL in 0.1 M HCl) at 4 °C for 48–72 h, neutralized to pH 7.4 with 1 M NaOH, and adjusted to 10 mg/mL. The hydrogel undergoes thermosensitive gelation at 37 °C, forming a stable scaffold in situ.

Pre-clinical biocompatibility was evaluated using the CCK-8 assay with RPE-1 cells cultured on dAM hydrogel for 24 and 48 h, with Corning^®^ Matrigel^®^ Matrix as a non-toxic reference. Surface cell viability was 59.61 ± 1.97% at 24 h and 56.40 ± 5.96% at 48 h, with no significant difference from Matrigel (64.59 ± 5.89%; p = ns). A transwell invasion assay confirmed active RPE-1 migration into the scaffold, indicating cell ingrowth rather than cytotoxicity, supporting biocompatibility prior to clinical use.

### Patient and preoperative assessment

A 62-year-old man with a history of diabetes mellitus (DM) and hypertension (HT) presented to the vitreoretinal clinic at Walailak University Hospital with progressive central visual distortion and decreased acuity in the right eye. Preoperative BCVA was 20/200 (logMAR 1.0) in the right eye with pinhole improvement to 20/125, and 20/80 in the left eye. IOP by iCare rebound tonometry was 9.7 mmHg (OD) and 10.5 mmHg (OS). Axial length was 23.0 mm.

**Fig. 1:**
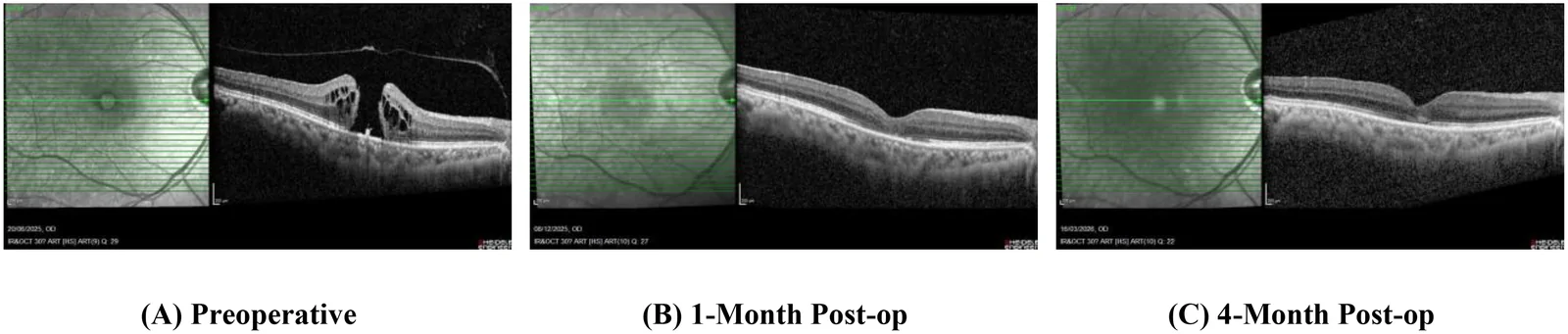
SD-OCT (Heidelberg Spectralis) timeline. (**A**) Preoperative: Gass stage 4 full-thickness macular hole with minimum diameter 572 μm and prominent intraretinal cystic changes. (**B**) One-month postoperative: complete Type 1 closure with restoration of foveal contour; dAM hydrogel no longer visible. (**C**) Four-month postoperative: maintained Type 1 closure with progressive ellipsoid zone recovery. Scale bar = 200 μm

SD-OCT (Heidelberg Spectralis, Heidelberg Engineering, Germany) confirmed a Gass stage 4 idiopathic FTMH with MD 572 μm, prominent intraretinal cystic changes, and a complete posterior vitreous detachment (PVD) (Fig. 1A). Ultra-wide-field fundus photography (Optos Daytona) showed no peripheral retinal pathology (Fig. 3A). Dilated fundus examination confirmed no diabetic retinopathy in either eye. Written informed consent was obtained in accordance with Walailak University IRB No. WUEC-24-331-01.

### Surgical technique: step-by-step hydrogel delivery

Surgery was performed on 5 November 2025 under retrobulbar anesthesia. Combined phacoemulsification (IOL AR40e, 18.0 D) and 25-gauge PPV were performed. The ILM was stained with 0.05% Heavy Brilliant Blue G dye (Ocublue Plus, Aurolab, India) mixed with 10% dextrose (1:2) under BSS and peeled circumferentially for ~ 2 disc diameters using 25-gauge end-gripping forceps (Grieshaber Asymmetrical Forceps, Alcon).

**Fig. 2:**
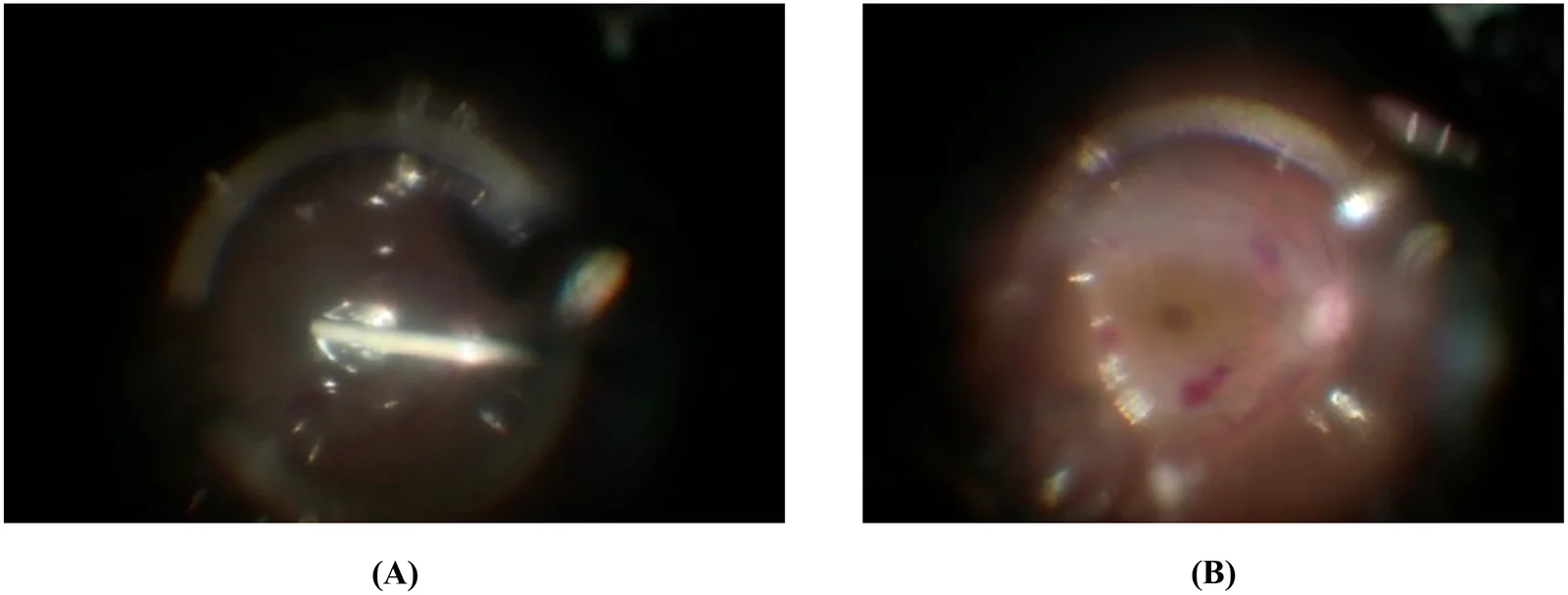
Intraoperative photographs. (**A**) dAM hydrogel injection via 25-gauge soft-tip cannula following ILM peeling and fluid-air exchange; hydrogel extends approximately 2 disc diameter from the fovea. (**B**) Macula after hydrogel placement, with the dAM scaffold in situ prior to SF6 instillation

Following complete ILM peeling, a fluid–air exchange was performed. The dAM hydrogel was then delivered onto the macular hole. Because the formulation is of low viscosity and is used in a very small volume (0.05–0.1 mL), it is dispensed not as a viscous bolus but as one to two discrete drops from a 25-gauge soft-tip cannula (Alcon) introduced through a valved trocar. The drops were placed directly over the macular hole under air. Owing to its fluid nature before gelation, the hydrogel naturally flowed into and filled the macular hole defect while a portion remained over the surrounding retinal surface, producing a continuous scaffold that both fills the hole and covers its edges. Importantly, the hydrogel was not inserted beneath the neurosensory retina into the subretinal space; it filled the defect and overlay the retinal surface without manipulation of the hole margins. The resulting coverage spanned approximately two disc diameters centred on the fovea (Fig. 2A and B). No instrument contact with the retinal surface was required at any point, avoiding the iatrogenic trauma associated with forceps-assisted scaffold placement and the potential retinal pigment epithelium injury associated with subretinal plug insertion.

A practical advantage of this technique is intraoperative confirmation of scaffold position. Although the hydrogel is optically transparent and cannot itself be directly visualised, waiting approximately 5–10 s after delivery reveals a subtle, slightly whitish reaction of the underlying retina in the area covered by the gel. This reaction, which persists for a period intraoperatively, serves as a reliable visual marker confirming both the location and the extent of scaffold coverage, and indicates that in-situ gelation at body temperature has begun. We regard this whitish tissue reaction as the principal intraoperative endpoint of correct hydrogel placement.

Once correct placement was confirmed, one milliliter of pure SF6 gas was injected. To minimise the risk of hydrogel displacement—the principal intraoperative pitfall shared by all macular-hole scaffold techniques—the fluid–air exchange and gas instillation were performed gently and away from the macular hole, and direct fluid currents over the gel were avoided. Sclerotomies were sutured with Vicryl 8 − 0 and the patient was placed prone. Total operative time was approximately 45 min.

#### Surgical pearls

Several technical points facilitate reproducible delivery. First, the small volume (0.05–0.1 mL, one to two drops) should be dispensed slowly and precisely over the centre of the hole; over-delivery risks spread beyond the intended macular area. Second, the fluid nature of the hydrogel allows it to fill the hole defect and cover the surrounding retina in a single step, without the need to insert material into the subretinal space; this avoids the retinal pigment epithelium trauma and parafoveal atrophy that have been associated with subretinal plug placement. Third, the no-touch delivery avoids forceps manipulation of the fragile hole margins. Fourth, surgeons should allow 5–10 s and look for the confirmatory whitish retinal reaction before proceeding. Finally, a slow, controlled fluid–air exchange directed away from the fovea, followed by short-acting gas tamponade and face-down positioning, protects the gel during the critical early gelation and adhesion phase.

### Postoperative course and outcomes

The patient maintained strict face-down positioning for 5 days postoperatively and received standard topical antibiotic and steroid drops.

#### One-week follow-up (13 November 2025)

No intraocular inflammation, hypopyon, or fibrinous reaction was observed; gas occupied approximately 70% of the vitreous cavity.

#### One-month follow-up (8 December 2025)

BCVA improved to 20/80 (logMAR 0.60), a 2-line Snellen gain. IOP was 16.4 mmHg. SD-OCT demonstrated complete Type 1 anatomical closure with restored foveal contour (Fig. 1B). No residual subretinal fluid, intraretinal cysts, or epiretinal membrane was observed. The dAM hydrogel was no longer visible on OCT, consistent with ECM biodegradation.

#### Four-month follow-up (16 March 2026; day 121)

**Fig. 3:**
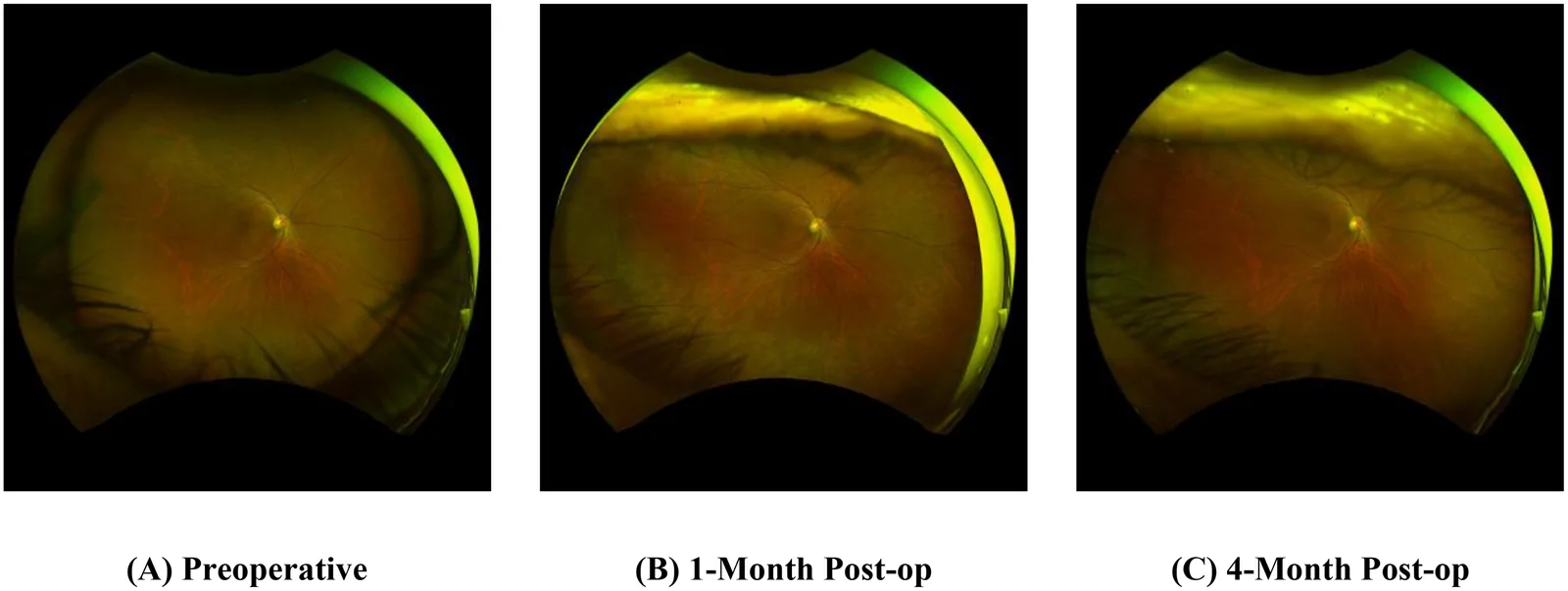
Ultra-wide-field fundus photography (Optos Daytona). (**A**) Preoperative: macular hole visible in the right eye; no peripheral retinal pathology. (**B**) One-month postoperative: macular hole resolved; flat, attached retina. (**C**) Four-month postoperative: maintained closure with well-attached retina and no peripheral complications

The patient reported no metamorphopsia. BCVA further improved to 20/40 + 1 (logMAR 0.30), a cumulative 3-line Snellen gain. IOP was 12.7 mmHg. Anterior segment examination showed clear cornea, no anterior chamber cells, and an in-place IOL. SD-OCT (Fig. 1C) confirmed sustained Type 1 closure with progressive EZ reconstitution and normal foveal contour. Ultra-wide-field fundus photography (Fig. 3C) confirmed no peripheral complications.

### Multifocal ERG

**Fig. 4:**
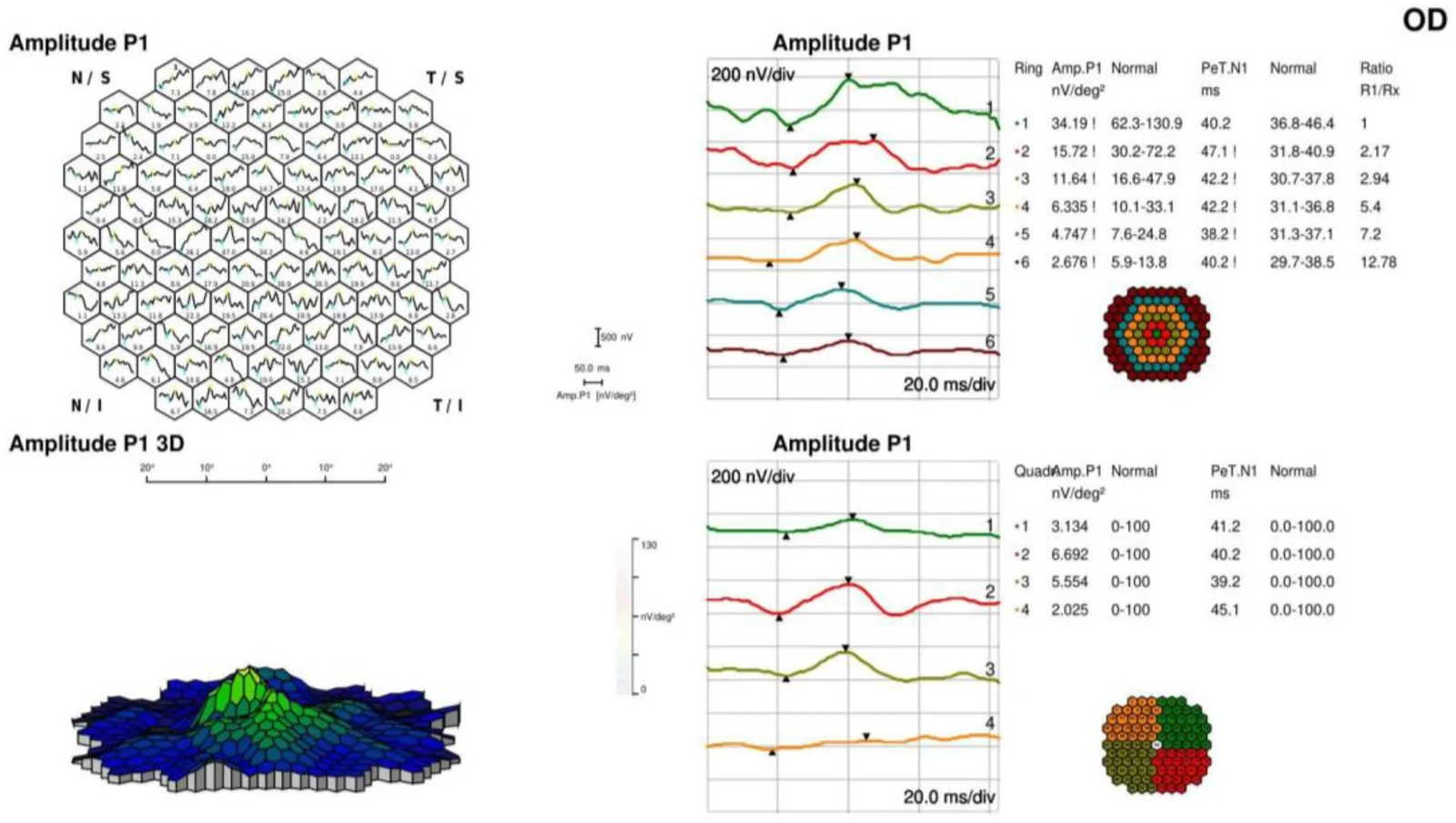
Multifocal ERG (mfERG; RETI-scan, 103 segments, Roland Consult) at four months (16 March 2026), right eye. Amplitude topographic map (upper left) and 3D plot (lower left) show a discernible foveal peak (Ring 1: 34.19 nV/deg²). Ring trace array (upper right) demonstrates measurable P1 responses across all six eccentricity rings. The central N1 implicit time (40.2 ms) is within the normal range (36.8–46.4 ms), confirming preserved foveal photoreceptor signal conduction at the former macular hole site. All ring amplitudes are reduced relative to normative values, consistent with expected deficits from a large chronic preoperative macular hole

mfERG (RETI-scan, 103 segments, Roland Consult, Germany; 28° view angle, DTL electrode, pupil 7 mm dilated) was performed at 4 months (Fig. 4). P1 amplitudes were reduced across all six rings relative to normative values, expected following a large chronic FTMH. Critically, the Ring 1 (foveal) N1 implicit time (40.2 ms) was within the normal range (36.8–46.4 ms), confirming preserved foveal photoreceptor signal conduction at the former macular hole site. Measurable responses were present across all rings with no electrophysiological evidence of intraocular hydrogel toxicity. Clinical and functional outcomes across all time points are summarized in Table 1.

### Summary of clinical and functional outcomes

**Table 1:** Summary of clinical and functional outcomes

Parameter	Preoperative	1 Month	4 Months
BCVA (Snellen / logMAR)	20/200 / 1.0	20/80 / 0.60	20/40 + 1 / 0.30
IOP OD (mmHg)	9.7	16.4	12.7
Macular hole status	Open (MD 572 μm, Gass 4)	Type 1 closure	Type 1 closure (maintained)
Ellipsoid zone (OCT)	Disrupted	Improved	Progressive recovery
Foveal contour (OCT)	Absent	Restored	Normal
Metamorphopsia	Present	Absent	Absent
AC cells	N/A	None	None
dAM hydrogel on OCT	N/A	Not visible	Not visible
mfERG R1 P1 (nV/deg²)	N/A	N/A	34.19
mfERG R1 N1 implicit time (ms)	N/A	N/A	40.2 (normal 36.8–46.4)
Adverse events	None	None	None

## Discussion

We describe an injectable dAM hydrogel scaffold technique for the surgical management of large idiopathic FTMH. The principal advantage of this technique lies in its delivery: a thermosensitive hydrogel that is injected through a standard 25-gauge cannula, conforms to hole geometry, and gels in situ at body temperature, thereby avoiding the manipulation required by solid scaffolds. To our knowledge this is the first reported intraocular application of such a hydrogel. In the illustrative case, the technique achieved sustained complete Type 1 anatomical closure at both one and four months, with progressive BCVA improvement from 20/200 to 20/40 + 1 (a cumulative 3-line Snellen gain) and objective electrophysiological confirmation of photoreceptor viability by mfERG, without any intraocular adverse events.

The biological rationale is grounded in the established properties of the amniotic ECM. Decellularization preserves structural proteins (collagen I, III, IV; laminin; fibronectin) and bioactive molecules—including anti-inflammatory [6] and anti-fibrotic [7] factors—that promote cell migration, proliferation, and tissue regeneration [9]. The hydrogel form confers critical intraocular advantages: injectability through 25-gauge cannulas, precise in-hole delivery, and thermosensitive in-situ gelation at 37°C. A distinctive feature of the fluid hydrogel is that, on delivery, it simultaneously fills the hole defect and covers the surrounding retinal surface, forming a single continuous scaffold without the need for subretinal insertion. This contrasts with the solid hAM plug, which is positioned in the subretinal space and may traumatise the retinal pigment epithelium, and with autologous ILM flaps, which require meticulous manipulation. By achieving both gap-filling and epiretinal coverage in one atraumatic step, the technique may combine the mechanical advantages of a plug with the reduced surgical trauma of a surface scaffold.

The four-month mfERG findings provide objective functional corroboration absent from prior FTMH biologic scaffold reports. The foveal N1 implicit time of 40.2 ms—within the normal range of 36.8–46.4 ms—indicates that surviving foveal photoreceptors retain adequate response kinetics. The absence of electrophysiological deterioration provides direct in vivo evidence against dAM hydrogel-related intraocular toxicity, corroborating the pre-clinical CCK-8 biocompatibility data.

Compared with the inverted ILM flap, dAM hydrogel injection avoids meticulous flap manipulation and iatrogenic foveal trauma risk. Unlike the solid hAM patch, the hydrogel is delivered through the standard surgical workflow without additional instruments. The biodegradation of the hydrogel—confirmed by its absence on OCT at one month—is a desirable property for any intraocular biomaterial.

This report has inherent limitations as a single-case observation without a preoperative mfERG baseline. The absence of baseline electrophysiology limits direct functional comparison; however, the postoperative mfERG findings are interpretable in the context of expected deficits from a large chronic FTMH.

A second limitation concerns the inability to directly visualize the dAM hydrogel in vivo, given its optical transparency and refractive index similar to vitreous—the hydrogel could not be discerned on clinical examination or OCT at any postoperative time point. Consequently, we cannot definitively exclude a contribution from the natural tissue bridge formation that can follow ILM peeling alone. Indirect support for a scaffold-mediated contribution comes from our pre-clinical transwell assays, which demonstrated active RPE-1 migration into the three-dimensional ECM scaffold. ECM degradation products may additionally provide paracrine trophic support to photoreceptors and Müller cells [9]. Intraoperative OCT or fluorescently labelled hydrogel constructs could provide more direct evidence of scaffold persistence and degradation kinetics in vivo.

A third limitation is the absence of interpretable early postoperative OCT. SD-OCT performed on day 1 was non-diagnostic because the residual SF6 gas bubble (~ 70% fill) produced complete posterior shadowing—an intrinsic limitation of gas-filled eyes rather than a gap in clinical assessment. Consequently, the early sequence of hydrogel biodegradation and edge approximation before confirmed closure at one month could not be documented. Serial early OCT (from day 7, after partial gas resorption) is a pre-specified endpoint of the ongoing MACROHOLE RCT.

Finally, we acknowledge the question of case selection. The presented patient had a Gass stage 4 FTMH with MD 572 μm and prominent intraretinal cystic changes with a substantial cuff height on SD-OCT. While cuff height may be associated with residual tangential traction, large holes of this diameter (MD > 500 μm) with Gass stage 4 morphology and complete PVD have reported primary closure rates of approximately 56–60% with conventional ILM peeling alone [2, 3], placing this case firmly in the high-risk surgical stratum for which adjunctive scaffold strategies are justified. For this first-in-human application, a technically straightforward case was intentionally selected—after IRB approval (WUEC-24-331-01) and informed consent for an experimental procedure—to minimize confounding and maximize interpretability of the safety signal. Defining the specific niche in which dAM hydrogel adds value over established techniques, including in refractory, recurrent, or extremely large FTMH, is a primary objective of the ongoing MACROHOLE RCT (NCT06433284).

## Conclusions

The injectable dAM hydrogel scaffold technique offers a simple, instrument-sparing method for delivering a biologic scaffold to large FTMH through the standard vitrectomy workflow. In this first-in-human application the technique achieved stable Type 1 closure with progressive visual improvement to 20/40 + 1 and electrophysiological recovery over four months, with mfERG evidence of preserved foveal photoreceptor function and no intraocular adverse events. These findings support the technique as feasible and safe, and justify prospective comparative evaluation against established scaffold methods.

## Data Availability

The datasets supporting the conclusions of this article are included within the article. Deidentified clinical data are available from the corresponding author upon reasonable request.
